# Use of illicit and prescription drugs for cognitive or mood enhancement among surgeons

**DOI:** 10.1186/1741-7015-11-102

**Published:** 2013-04-09

**Authors:** Andreas G Franke, Christiana Bagusat, Pavel Dietz, Isabell Hoffmann, Perikles Simon, Rolf Ulrich, Klaus Lieb

**Affiliations:** 1 Department of Psychiatry and Psychotherapy, University Medical Centre Mainz, Untere Zahlbacher Str. 8, Mainz, 55131, Germany; 2 Department of Sports Medicine, Rehabilitation and Disease Prevention, Faculty of Social Science, Media and Sports, Johannes Gutenberg-University Mainz, Albert-Schweitzer-Str. 22, Mainz, 55128, Germany; 3 Institute of Medical Biostatistics, Epidemiology and Informatics (IMBEI), University Medical Centre Mainz, Obere Zahlbacher Str. 69, Mainz, 55131, Germany; 4 Department of Psychology, University of Tübingen, Schleichstr. 4, Tübingen, 72076, Germany

**Keywords:** Cognitive enhancement, Surgeon(s), Prescription drug(s), Stimulant(s)

## Abstract

**Background:**

Surgeons are usually exposed to high workloads leading to fatigue and stress. This not only increases the likelihood of mistakes during surgery but also puts pressure on surgeons to use drugs to counteract fatigue, distress, concentration deficits, burnout or symptoms of depression. The prevalence of surgeons taking pharmacological cognitive enhancement (CE) or mood enhancement (ME) drugs has not been systematically assessed so far.

**Methods:**

Surgeons who attended five international conferences in 2011 were surveyed with an anonymous self-report questionnaire (AQ) regarding the use of prescription or illicit drugs for CE and ME and factors associated with their use. The Randomized Response Technique (RRT) was used in addition. The RRT guarantees a high degree of anonymity and confidentiality when a person is asked about stigmatizing issues, such as drug abuse.

**Results:**

A total of 3,306 questionnaires were distributed and 1,145 entered statistical analysis (response rate: 36.4%). According to the AQ, 8.9% of all surveyed surgeons confessed to having used a prescription or illicit drug exclusively for CE at least once during lifetime. As one would expect, the prevalence rate assessed by RRT was approximately 2.5-fold higher than that of the AQ (19.9%; 95% confidence interval (CI), 15.9% to 23.9%, N = 1,105). An even larger discrepancy between the RRT and AQ was observed for the use of antidepressants with a 6-fold higher prevalence (15.1%; 95% CI, 11.3% to 19.0%, N = 1,099) as compared to 2.4% with the AQ. Finally, logistic regression analysis revealed that pressure to perform at work (odds ratio (OR): 1.290; 95% CI, 1.000 to 1.666; *P* = 0.05) or in private life (OR: 1.266; 95% CI, 1.038 to 1.543; *P* = 0.02), and gross income (OR: 1.337; 95% CI, 1.091 to 1.640; *P* = 0.005), were positively associated with the use of drugs for CE or ME.

**Conclusions:**

The use of illicit and prescription drugs for CE or ME is an underestimated phenomenon among surgeons which is generally attributable to high workload, perceived workload, and private stress. Such intake of drugs is associated with attempts to counteract fatigue and loss of concentration. However, drug use for CE may lead to addiction and to overestimation of one’s own capabilities, which can put patients at risk. Coping strategies should be taught during medical education.

## Background

Surgeons are often exposed to an excessive workload leading to mental and physical exhaustion, for example, fatigue, sleep deprivation (especially in shift work), burnout, and even to higher rates of suicide [1-5]. This increases the likelihood of mistakes during surgery [6-9]. In order to maintain high cognitive performance, surgeons can come under pressure to counteract fatigue, distress, concentration deficits, burnout or symptoms of depression by the use of enhancing substances. Warren and colleagues discussed possible reasons for surgeons to consider the use of substances for cognitive enhancement (CE) or mood enhancement (ME), including such issues as internal (to maximize one´s own potential) and external pressure (by the employer or the public), patient safety or surgeons’ well-being [10]. However, there is no scientific evidence so far that either external or internal pressure leads to the consumption of prescription or illicit drugs to cope with these stressors. Nevertheless, performance enhancing drugs may be especially attractive to surgeons due to the fact that they appear to be a time-saving and, to some extent, an effective and easy alternative to more time-consuming coping strategies (for example, napping, sleep, relaxation techniques, and so on) or the use of coffee which may cause tachycardia and worsening tremor [10,11].

Thus, the use of prescription and illicit drugs could be a coping strategy to manage poor working conditions. However, (psycho-) stimulants (amphetamines (AMPH), methamphetamine, methylphenidate (MPH)), modafinil as well as antidementives and antidepressants have no consistent effects for CE or ME in healthy non sleep-deprived subjects [12-20]. Nevertheless, reduction of cognitive performance due to sleep deprivation is a common problem in shift work. Stimulants and modafinil have been demonstrated to attenuate disruption in cognitive performance and mood during night-shift work and sleep deprivation [20-25]. Previous studies revealed the use of stimulants and modafinil for CE purposes among students having at least an immediate pro-vigilant effect [14,26,27]. Beyond that, antidepressants such as selective serotonin reuptake inhibitors (SSRI) are known to be used for ME, although previous studies showed no immediate or delayed mood enhancing effect [18,28,29]. Furthermore, it is unclear whether ME affects only mood or also other aspects such as self-esteem or self-representation.

Previous studies among high school and university students using anonymous questionnaires (AQ) have shown prevalence rates for the use of prescription stimulants (MPH) to be 0.8% and 2.9% for illicit stimulants (AMPH) whereas 10.5% decided to use caffeine tablets for CE [27,30,31]. Furthermore, an online poll conducted by the journal *Nature* depicts a lifetime prevalence rate of 20% for stimulants, modafinil or beta blockers for CE purposes among participating academics [32]. A recent study using the Randomized Response Technique (RRT) shows prevalence rates of even 20% for the use of prescription and illicit drugs among university students for CE [33]. Studies using RRT guarantee an especially high degree of privacy, anonymity, and confidentiality when a person is prompted to answer sensitive questions about socially undesirable or illicit behavior [34-38].

This present survey reports the first data on prescription and illicit drug use for CE and ME among surgeons, together with usage association factors. We estimated prevalence rates of CE and ME among surgeons using AQ and RRT.

## Methods

The data for this study resulted from a survey conducted in 2011 among 3,306 German-speaking surgeons who attended five international conferences of the German Society of Surgery (Deutsche Gesellschaft für Chirurgie). After the first conference, potential participants were asked if they had been assessed before; those who had been were excluded from a second participation.

Based on previous research about prescription and illicit drugs for CE, an AQ about the use of prescription and illicit drugs for enhancing cognitive functions or mood was developed and distributed to the participants during the conferences. The AQ asked about the ‘non-medical use of stimulants with the particular intention of CE’ and ‘the non-medical use of antidepressants with the particular intention of ME’ during lifetime, last year, last month and last week (frequency). Furthermore, we asked for the age of first use. Beyond that, the AQ included questions about potential risk factors associated with usage of drugs and questions about biometrical parameters (for example, gender, age, age of first use, and so on.). We were mainly interested in healthy participants using drugs specifically for CE or ME. Therefore, the data of participants with self-reported psychiatric disorders (for example, depression, attention deficit/hyperactivity disorder, (ADHD)) who had physicians’ prescriptions for any drug were excluded.

Participants were asked to drop the questionnaire into black boxes after having filled in the questionnaire anonymously.

The study was carried out according to the Principles for Medical Research Involving Human Subjects according to the Declaration of Helsinki. The study was approved by the local Ethics Committee (Landesärztekammer Rheinland-Pfalz) (No. 837.321.08 (6318)). Participants gave informed consent by returning the questionnaire and were informed about this procedure in the introduction section of the questionnaire; this procedure was approved by the above mentioned local Ethics Committee.

The AQ contained questions about the use of drugs for CE and ME as well as questions using RRT. After a brief introduction about the RRT stressing the anonymity of this technique, questions were presented to participants as follows:

Please consider a certain birthday (yours, your mother’s, etc.). Is this birthday in the first third of a month (1st to 10th day)?

If yes, please proceed to Question A; if no, please proceed to Question B.

Question A: Is this birthday in the first half of the year (prior to the first of July)?

Question B: Did you ever use prescription and/or illicit drugs (e.g. Methylphenidate, Modafinil, illicit Amphetamines, and so on) without a medical need for cognitive enhancement?

Note that only you know which of the two questions you will answer

o Yes o No

For assessing the use of antidepressants for mood enhancement, we modified Question B as follows: ‘Did you ever use antidepressants without medical need for enhancing your mood and/or self-esteem, self-presentation?’

The interviewers are not able to know which question the respondent has to answer. Therefore, participants can reply honestly without compromising themselves. Of all participants, 67.1% (245.25/365.25) received the sensitive question (B) and 32.9% (120/365.25) the non-sensitive question (A).

$${\hat{\pi }}_{s}=\frac{a-\left(1-p\right)\;\pi_{N}}{p}$$

the proportion of ‘yes’ responses with respect to the sensitive questions can be estimated from proportion *a* of total ‘yes’ responses in the sample. *p* denotes the probability of receiving the sensitive question (Question B; p = 67.1% of all participants received this question). The probability of answering the non-sensitive question (A) with ‘yes’ is *π*_*n*_ = 49.6% (181.25/365.25). A 95% confidence interval (CI) for the unknown prevalence can be computed from the sampling variance

$$\mathrm{Var}\left({\hat{\pi }}_{s}\right)=\frac{a\;\left(1-a\right)}{n\;p^{2}}$$

where *n* denotes the sample size [33,39].

$$\begin{array}{l} \begin{array}{l} \mathrm{For}\;\mathrm{example}\;\mathrm{for}\;a=328/1105\approx 0.30,\\ \;p=245.25/365.25\approx 0.67,\\ \;\pi_{N}=181.25/356.25\approx 0.50,\;\mathrm{and}\\ \;n=1105,\;\mathrm{this}\;\mathrm{yields} \end{array}\\ {\hat{\pi }}_{s}=\frac{328/1105-\left(1-245.25/365.25\right)\;181.25/365.25}{245.25/365.25}\\ \;\approx 0.1993=19.93\%.\\ {\mathrm{Var}(\hat{\pi }}_{s})=\frac{328/1105\;\left(1-328/1105\right)}{1105\;{\left(245.25/365.25\right)}^{2}}\approx 0.000419.\\ {\mathrm{SE}(\hat{\pi }}_{s})=\sqrt{0.000419}\approx 0.0205=2.05\%.\\ \begin{array}{l} \mathrm{Thus}\;\mathrm{the}\;95\%\;\mathrm{confidence}\;\mathrm{interval}\;\mathrm{is}\;19.93\pm \;1.96\;2.05,\;\mathrm{that}\;\mathrm{is},\;\mathrm{ranges}\;\mathrm{from}\\ 15.91\%\;\mathrm{to}\;23.95\%. \end{array} \end{array}$$

Statistical analyses were performed with SPSS for Windows, Version 17.0. Means are given with their corresponding standard deviation (SD) (mean ± SE) and Clopper-Pearson confidence intervals (95% CI). AQ questions were analyzed using a multiple logistic regression analysis. For the regression, the variable selection procedure was performed by using stepwise forward selection with a selection level of 0.05. The variables which were analyzed as potential multivariable predictors of the use of prescription or illicit drugs for CE before forward selection were (available parameters): pressure to perform at work, pressure in private life, gross income, gender, age, family status, living with children, type of employer, employment status, hours of work, satisfaction with professional success, evaluation of career opportunities, pressure to perform subjectively evaluated as burdensome and pressure to perform subjectively evaluated as harmful to health. Ordinal variables with five or more categories (pressure to perform at work, pressure in private life, gross income, pressure to perform subjectively evaluated as burdensome and pressure to perform subjectively evaluated as harmful to health) were treated as continuous variables. Table 1 shows the variables included for the regression after forward selection; all variables which significantly influence the drug use for CE/ ME are listed in this table. There are no further variables for which we adjust. The results are presented as odds ratio (ORs) with confidence limits and *P*-values. The regression has been analyzed by referring to cases without missing values (complete case analysis).

**Table 1:** Risk factors for the use of prescription or illicit drugs for CE among surgeons by multivariable analysis

**Parameters**	**OR**	**95 % confidence limits**		***P*-value**
Pressure to perform at work	1.327	1.010	1.743	0.042
Pressure to perform in private life	1.252	1.015	1.543	0.036
Gross income	1.406	1.133	1.744	0.002

## Results

### Participants´ characteristics

A total of 1,204 (36.4%) of 3,306 distributed questionnaires were returned. Of the participants, 61 had to be excluded: 9 for not being a physician, 11 for having a physician`s prescription for drugs because of mental disorders and 39 (pair wise) for giving incomplete answers. Thus, the data of 1,145 surgeons entered the final statistical analysis. The mean weekly workload was estimated to be 56.8 ± 13.0 hours. Of the respondents, 56.4% were surgeons in training, 21.5% senior surgeons and 22.1% directors or deputy directors.

The pressure to perform optimally at the job was estimated to be severe (3.3 ± 1.2 on a 6-point Likert scale, 0 = not at all, 5 = very much), judged to be moderate to severely burdensome (2.6 ± 1.3) and moderate to severely harmful (2.7 ± 1.3). For further details, see Table 2.

**Table 2:** Participants` characteristics

**Participants (total)**	**N = 1,145 (= 100%)**
Gender	
Male	797 (69.7%)
Female	346 (30.3%)
Age	
Years (Mean ± SD)	24 to 85 years (43.30 ± 10.67)
Family status:	
Married	875 (76.5%)
Divorced	67 (5.9%)
Single	189 (16.5%)
Widowed	13 (1.1%)
Children:	
Participants living with children	525 (48.0%)
Participants living without children	568 (52.0%)
Type of employer:	
University	315 (27.7%)
Hospital (other than University hospital)	636 (56.0%)
Doctor´s office/ Doctor´s surgery	159 (14.0%)
Other (for example, industry)	26 (2.3%)
Employment status:	
Surgeons in training (1st to 5th year)	646 (56.4%)
Senior surgeons	246 (21.5%)
Directors/deputy directors	253 (22.1%)
Hours of work (per week)	
Hours (Mean ± SD)	13 to 100 (56.77 ± 12.97)
Gross income:	
<40,000 €	64 (5.7%)
40,000 to 100.000 €	544 (48.6%)
100,000 to 150.000 €	298 (26.6%)
150,000 to200.000 €	102 (9.1%)
>200,000 €	111 (9.9%)
Satisfied with professional success	
Yes	975 (87.8%)
No	136 (12.2%)
Subjective evaluation of career opportunities	
Mean ± SD:	2.49 ± 0.94
	1 = 126 (11.5%)
	2 = 488 (44.7%)
	3 = 342 (31.3%)
	4 = 91 (8.3%)
	5 = 44 (4.0%)
Pressure to perform on the job	
Mean ± SD:	3.32 ± 1.18
	0 = 32 (2.8%)
	1 = 67 (5.9%)
	2 = 131 (11.5%)
	3 = 318 (27.9%)
	4 = 452 (39.7%)
	5 = 138 (12.1%)
Pressure to perform in private life	
Mean ± SD:	2.01 ± 1.26
	0 = 140 (12.4%)
	1 = 267 (23.7%)
	2 = 329 (29.2%)
	3 = 246 (21.9%)
	4 = 118 (10.5%)
	5 = 251 (2.2%)
Pressure to perform subjectively evaluated as burdensome	
Mean ± SD:	2.57 ± 1.28
	0 = 62 (5.5%)
	1 = 196 (17.3%)
	2 = 260 (22.9%)
	3 = 323 (28.4%)
	4 = 238 (21.0%)
	5 = 57 (5.0%)
Pressure to perform subjectively evaluated as harmful to health	
Mean ± SD:	2.72 ± 1.34
	0 = 71 (6.3%)
	1 = 158 (14.0%)
	2 = 233 (20.6%)
	3 = 299 (26.4%)
	4 = 292 (25.8%)
	5 = 78 (6.9%)

Use of 6-point Likert scale, 0 = not at all, 5 = very much in respect of the following items: Subjective evaluation of career opportunities; Pressure to perform; Subjective pressure to perform. Means are given with standard deviation (SD).

Prevalence rates of cognitive enhancement (CE) and mood enhancement (ME) measured by anonymous questionnaires (AQ).

Lifetime-prevalence of CE and ME is higher than last-year prevalence, which in turn is higher than last-month and last-week prevalence rates (see Table 3 and 4). Differences between last-year and last-month prevalence rates for CE and ME are small, whereas the difference between lifetime prevalence and last-year prevalence rate is remarkably higher. Age of first use did not differ significantly between prevalence rates. For more details on lifetime-, last-year, last-month and last-week prevalence rates, as well as for age of first use using the AQ, see Tables 3 and 4.

**Table 3:** AQ results for prevalence rates of the use of prescription drugs + illicit drugs for CE

**Prescription drugs + illicit drugs**	**N**	**%**	**Age (mean ± SD) of first use**
Last Week	8	0.84	25.00 ± 1.41
Last Month	13	1.36	24.40 ± 1.44
Last Year	29	3.03	24.47 ± 8.04
Lifetime	85	8.88	23.99 ± 6.21

Illicit drugs: Ecstasy, ephedrin, cocaine, illicit AMPH. AMPH, amphetamines; CE, cognitive enhancement; AQ, anonymous questionnaire; N, number.

**Table 4:** AQ results for prevalence rates of the use of antidepressants for ME

**Prescription drugs + illicit drugs**	**N**	**%**	**Age (mean ± SD) of first use**
Last Week	4	0.42	26.00 ± 0
Last Month	5	0.52	30.00 ± 5.66
Last Year	10	1.04	34.67 ± 9.02
Lifetime	23	2.40	38.67 ±10.65

AQ, anonymous questionnaire; ME, mood enhancement; N, number.

### Prevalence rates of CE and ME measured by AQ compared to the randomized response technique (RRT)

Prevalence rates measured by the RRT are considerably higher than prevalence rates measured by AQ. Table 5 shows that with AQ, 8.9% of the surgeons confessed to having used a prescription or illicit drug exclusively for CE at least once during their lifetime. In contrast, the corresponding RRT estimate was approximately 2.5-fold higher than the AQ estimate, that is, 19.9% (95% CI, 15.9% to 23.9%, n = 1,105). An even larger discrepancy between the RRT and AQ was observed for the use of antidepressants with a 6-fold higher prevalence rate, that is, 15.1% (95% CI, 11.3% to 19.0%, n= 1,099) as compared to 2.4% with the AQ.

**Table 5:** AQ and RRT results for lifetime prevalence rates of prescription or illicit drug use for cognitive enhancement (CE) or mood enhancement (ME)

	**Study technique**		**Age of first use of enhancement**
**Substance use for CE/ ME:**	**AQ**	**RRT**	**(mean ± SD)**
	**(n= 957)**	**(n= 1,102)**	**(according to AQ)**
Any prescription or illicit drug	8.9% (n = 85)	19.9%	24.0 ± 6.2
Methylphenidate (MPH)	2.5% (n = 24)		22.0 ± 2.8
Amphetamine pills	2.6% (n = 25)		22.6 ± 3.6
Illicit amphetamines	0.9% (n = 9)		23.0 ± 2.0
Modafinil	2.2% (n = 21)		35.8 ± 7.2
Ecstasy	0.6% (n = 6)		20.2 ± 3.0
Cocaine	1.6% (n = 15)		22.7 ± 3.0
Ephedrin	1.2% (n = 11)		19.3 ± 6.0
Antidementive drugs	0.3% (n = 3)		25.0 ± 2.8
Atomoxetin	0.6% (n = 6)		26.0 ± 0.0
Any antidepressant	2.4 (n = 23)	15.1%	38.7 ± 10.7

Lifetime prevalence rates of prescription or illicit drug use for CE and antidepressants for ME among 1,143 surgeons by anonymous questionnaire (AQ) compared to the Randomized Response Technique (RRT). Means are given with standard deviation (SD).

### Factors associated with the use of prescription and illicit drugs for CE/ ME

Finally, a logistic regression analysis revealed that pressure to perform at work (OR: 1.327; 95% CI: 1.010 to 1.743; *P* = 0.042) or in private life (OR: 1.252; 95% CI: 1.015 to 1.543; *P* = 0.036) and gross income (OR: 1.406; 95% CI: 1.133 to 1.744; *P* = 0.002) were positively associated with the use of drugs for CE or ME (see Table 4).

Logistic regression analysis suggests that other factors play no role in the use of prescription and illicit drugs: gender (*P* = 0.809), age (*P* = 0.620), family status (*P* = 0.698), living with children (*P* = 0.720), type of employer (*P* = 0.151), employment status (*P* = 0.820), hours of work (*P*= 0.366), satisfaction with professional success (*P* = 0.829) and evaluation of career opportunities (*P* = 0.822).

## Discussion

The AQ results of this study indicate that 8.9% of all surveyed surgeons used prescription or illicit drugs with the particular intention of CE by AQ. By contrast, the RRT results showed a higher prevalence of 19.9%. Furthermore, using AQ, 2.4% answered that they had already used antidepressants for ME whereas the RRT revealed a prevalence of 15.1%. Furthermore, prescription or illicit drug use for CE or ME was associated with the pressure to perform at work or in private life and with gross income.

On the one hand, there are substantial differences regarding the prevalence rate in the present study. On the other hand, there are significant differences compared with previous studies of drug use for performance enhancement. There are an increasing number of studies dealing with ‘academic performance enhancement’, ‘cognitive enhancement’ or ‘pharmacological neuroenhancement’ regarding cognition (for example, [10,26,27,32,40-42]).

Regarding prevalence rates and associated factors, it is useful to consider several factors as follows: With the exception of the present study, there exists a severe paucity of data about drug use for CE among employed adults. DAK, a German health insurance company, online surveyed via e-mail 20,000 employed members (20- to 50-years old) with a response rate of 15%. Participants were asked about their use of various substances for CE and mental well-being without medical need [43]. Without accurately distinguishing prescription and over-the-counter drugs, the non-representative DAK study showed a lifetime prevalence rate of 5%. Stated reasons for usage were: ‘depressed mood’, ‘anxiety’, ‘nervousness’, ‘uneasiness’, ‘memory deficits’, ‘fatigue’, and ‘problems of concentration’ [43]. These rationales seem to be the same as among surgeons [1-5]. Furthermore, a non-random online poll by the journal *Nature* which unfortunately did not specify respondents, demonstrated that 20% of participants had already used prescription drugs for non-medical reasons to improve concentration and improve their focus for a specific task. MPH was the most popular substance, followed by modafinil and beta blockers [32]. MPH and modafinil are also the most prevalently used drugs in our survey. This agrees with the results of our study, although, admittedly, the surveyed groups are not directly comparable. Interestingly, these results match the RRT results of our study. Both surveys, online polls as well as the present RRT study, guarantee a relatively high level of anonymity. This may be one of the most important aspects when assessing pharmacological CE or ME, both potentially highly stigmatizing subjects.

Outside of these particular studies, only students’ substance use for academic performance enhancement has been surveyed. A previous study by our research group among 1,500 high school and university students (over 18 years) using AQ, assessed lifetime prevalence rates of 1.29% for prescription stimulants (MPH, AMPH) and 2.6% for illicit stimulants [27]. Regarding lifetime prevalence, we found that prevalence rates for stimulants in the present study are slightly higher than in our earlier students’ survey. This may be associated with the older age of surgeons.

Furthermore, both studies excluded participants with ADHD or other psychiatric disorders where prescribed psychiatric medications were involved. Many other studies did not exclude these ‘patients’, revealing higher prevalence rates including those where participants misuse their own prescribed medication [40].

A meta-analysis by Wilens and colleagues examining prevalence rates of stimulant misuse included 21 US studies with 113,000 participants revealing a past-year prevalence rate of 5% to 9% in grade and high schools and 5% to 35% in college-age individuals [40]. For this important meta-analysis which included many significant studies about stimulant misuse among students, CE is only a side aspect of the study. This explains the substantially higher (past-year) prevalence rate compared to the present study. However, ‘to concentrate’ and ‘improve alertness’ have been salient participants’ reasons for misuse of stimulants.

The most recent study about CE among 2,600 students using the RRT shows a one-year prevalence rate of 20% for the non-medical use of prescription and illicit drugs for CE [33]. These results show a comparable prevalence to those of the present study.

Beyond that, Partridge and colleagues revealed that a high percentage of the public media portrayed CE as common which accords with our high prevalence rate for CE [42]. However, this finding contrasts with that of our survey study of the same group among university students leading to the assumption of a ‘phantom debate’ [44,45].

While we were not able to show a significant influence of gender on the use of potential CE- or ME-substances, Dietz and colleagues revealed that significantly more male than female students used prescribed or illicit drugs for CE. Our results do not confirm this finding. The literature is somewhat inconsistent on this subject. For the illicit use of prescription ADHD medications among college students, DeSantis and colleagues found a significantly higher prevalence rate in male than in female students [46], whereas Teter and colleagues found no gender differences regarding prescription stimulant use among college students [47]. However, studies focusing upon this particular association in the context of a different surveyed group from that of the present study, suggest higher risk behaviors in male compared to female subjects [33,48].

Surveyed surgeons answered that their age of first use of prescription or illicit drugs for CE was 24.0 years. However, our previous study among 1,500 students revealed 17 to 18 years to be the age of first use of prescription or illicit drugs for CE [27]. This is almost 5 to 6 years younger than among surgeons, who themselves had been medical students and later trainee surgeons, decades before. However, study participants are 43 years old (mean) which may imply that two decades ago, the use of CE drugs started substantially later in life. Beyond that, first use of antidepressants for ME was 39 years (mean) compared to an average of 24-year-old participants using CE drugs for the first time.

Methodologically, all these studies only allow an indirect comparison of different survey methods. The present study allows us for the first time to compare AQ questions with RRT questions in one single integrated survey about drug use. In this respect, a previous meta-analysis of 38 RRT validation studies by Lensvelt-Mulders and colleagues reported that RRT provides more valid data than other survey methods. This strengthens the validity of the RRT prevalence rates of 19.9% for CE and 15.1% for ME [49]. This underlines the relevance of the survey method in general. In particular, it strengthens the validity and reliability of the higher RRT results of 19.9% and 15.1% for CE and ME compared to the lower prevalence rates using direct questions.

We were able to show that pressure to perform at work or in private life, together with gross income, are positively associated with the use of prescription or illicit drugs for CE or ME and are the only factors associated with drug use for this purpose. Further hypothesized factors were revealed to play no role in the use of prescription or illicit drugs for CE. We found a positive association of pressure to perform at work or in private life and gross income with the use of drugs for CE. However, we cannot interpret this finding as a general proof of a direct causal relationship between feeling pressure and the use of CE substances. Furthermore, this association is not tenable for professional life in general. Such factors should be addressed in detail in further studies. At least to our present knowledge, there are no data from empirical studies which allow meaningful comparison with our data: studies of students’ drug use focusing on CE as well as the previously cited *Nature* poll did not examine these factors.

Surgeons should know about the effects and side-effects of the substances used for CE or ME, at least regarding prescription drugs, such as methylphenidate, amphetamine tablets (for example, Adderall®), atomoxetine, modafinil, antidementive drugs and antidepressants. A survey study by Partridge and colleagues showed that university students already seem to have a realistic idea of the effects and side-effects [44]. According to randomized controlled trials (RCTs), reviews and meta-analyses there are almost no pro-cognitive effects regarding normal healthy non-sleep-deprived subjects on simple and higher cognitive domains [12-17].

Nevertheless, stimulants and modafinil have enhancing effects on simple cognitive domains, such as fatigue, vigilance, psychomotor skills and reaction time in sleep-deprived subjects; furthermore, there are slightly pro-cognitive effects on higher cognitive domains and, beyond that, stimulants have subjective ‘pro-effects’ [12,14,19-25]. One can presume that the effects on higher cognitive skills are indirect effects which are mediated via simple cognitive skills, for example, vigilance. The fact that sleep deprivation leads to clearer results supports this hypothesis [12].

One would expect surgeons to know these limited effects and to avoid the use of these prescription and illicit drugs for CE. However, every fifth surgeon has already used these drugs. We can only speculate about the reasons. On the one hand, surgeons may not know the missing pro-cognitive effects or overestimate the effects of such drugs. On the other hand, knowledge – and even overestimation – about pro-cognitive effects in sleep-deprived subjects only confirms that sleep deprivation is a common phenomenon among surgeons.

Beyond that, antidepressants (such as SSRI) have no mood enhancing effect in normal healthy subjects at all [12,18]. Nevertheless, 15% have already used antidepressants for ME, which may indicate missing knowledge about the effects of antidepressants in normal healthy subjects or overestimation of the putative effects.

Another important factor is the side-effect profile and safety risks of amphetamines which have to be considered. Beyond severe side-effects which are described in package-inserts accompanying these drugs and the results of RCTs, reviews and meta-analyses (for example, jitteriness, agitation, cardiologic side effects, such as tachycardia, hypertension, gastro-intestinal side effects, such as stomach ache, diarrhea, and so on), stimulants can cause addiction and further addictive behavior. Also, the misuse of illicit drugs and prescription drugs without prescription is a federal offense.

A number of limitations of the present study are worth identifying here. We obtained a response rate of 36.4% which is a low response rate compared to previous studies using RRT (for example, [27,33]). However, questionnaires were distributed during conference breaks in huge buildings, so that we were hardly able to control potential participants’ behavior concerning the return of the questionnaires to the black boxes provided. Furthermore, substance use – or even misuse – can be considered a highly stigmatizing subject leading to low response rates. Thus, a response rate of 36.4% may be considered relatively high and comparable to other studies assessing stigmatizing subjects with anonymous questionnaires [36]. However, the response rate of 36.4% together with the non-random sample limits the generalizability of our findings.

Another important factor is the likelihood of a participation bias: Since the response rate is only one third, we do not know in particular whether subjects with more positive attitudes or more negative attitudes on the topic participated disproportionately which may have caused a response bias. Since many more male subjects participated in our study, a potential gender bias exists. This may explain why we did not find gender differences in prevalence rates whereas earlier studies including our own have partly shown that male subjects more often used drugs for CE than female subjects.

Beyond that, we asked surgeons for the non-medical use of stimulants for CE and antidepressants for ME. However, we did not specifically ask for the context of use, for example, whether surgeons had used it directly prior to surgical interventions.

## Conclusions

The use of illicit and prescription drugs for CE or ME is an underestimated phenomenon among surgeons. The present results indicate that about 15% to 20% of surgeons have used drugs for CE or ME at least once during their lifetimes. This may be attributed to high workload and perceived work-related and private stress.

Substances such as modafinil seem to counteract fatigue and loss of concentration and thus may provide simple pharmacological help for stressed surgeons. However, pro-cognitive effects on higher cognitive domains are very limited. Furthermore, the side effects and effects of long-term use (for example, misuse, addiction) of such drugs seem to be underestimated by users. Beyond that, stimulant use may put patients at risk given the fact that previous research has shown that stimulants may lead to overestimation of one’s own capabilities. Both factors may be harmful for users. The contemporary debate on cognition-enhancing drugs requires a broader data base on consumption rates in populations at risk, together with careful studies of drug (side) effects to substantiate discussions of ethical and legislative aspects. Therefore, I) information about the restricted usefulness and risks of the use should be provided, II) guidelines on how to deal with drug use among employees who have contact with patients have to be provided, and III) information about, and the development of, relevant coping strategies has to become an integral part of medical education.

## Abbreviations

AMPH: Amphetamine(s); AQ: Anonymous questionnaire; ADHD: Attention deficit/ hyperactivity disorder; CE: Pharmacological cognitive enhancement; CI: Confidence interval; ME: Pharmacological mood enhancement; MPH: Methylphenidate; OR: Odds ratio; RCT: Randomized controlled trial; RRT: Randomized Response Technique; SD: Standard deviation; SSRI: Selective serotonin reuptake inhibitor

## Competing interests

The authors declare that they have no competing interests.

## Authors’ contributions

AGF, KL and PS participated in the conception and design of the study. AGF, KL and PS monitored data collection. AGF, KL, CB and in particular IH analyzed and checked the AQ data; PD, PS and RU analyzed the data of the Randomized Response Technique (RRT) which was cross-checked by IH. All authors participated in data interpretation, drafting, and revising the manuscript. All authors read and approved the final manuscript.

## Authors’ information

AGF, CB and KL belong to the Department of Psychiatry and Psychotherapy, University Medical Centre Mainz, Germany. KL and AGF are psychiatrists, CB is a sociologist. IH is a mathematician and expert in statistics belonging to the Institute of Medical Biostatistics, Epidemiology and Informatics (IMBEI) of the University Medical Center of the Johannes-Gutenberg University Mainz. PD and PS belong to the Department of Sports Medicine, Rehabilitation and Disease Prevention, Faculty of Social Science, Media and Sports, Johannes Gutenberg-University Mainz, Germany. PS is trained in internal medicine, expert in sports medicine and an active member of the National and World Anti Doping Agency (NADA and WADA). RU belongs to the Department of Psychology, University of Tübingen, Germany; he is trained in mathematical psychology and statistics and is a member of WADA’s doping prevalence research group.

## Pre-publication history

The pre-publication history for this paper can be accessed here:

http://www.biomedcentral.com/1741-7015/11/102/prepub
